# Atypical Femoral Fracture Secondary to Long-Term Bisphosphonate Use: A Case Report

**DOI:** 10.7759/cureus.90611

**Published:** 2025-08-20

**Authors:** Madhumati Mandal, Haiqah Amjad, Japheth Oyovwi, Aliyu O Olaniyi, Muhammad Fayyaz

**Affiliations:** 1 Internal Medicine, Stockport NHS Foundation Trust, Stockport, GBR; 2 General Medicine, Stepping Hill Hospital, Manchester, GBR; 3 Geriatrics Medicine, Stockport NHS Foundation Trust, Stockport, GBR

**Keywords:** atypical femoral fracture, atypical fracture, bisphosphonates, bone health, geriartrics, long-term therapy, multiple myeloma, osteopenia, osteoporosis, sodium clodronate

## Abstract

Atypical femoral fractures (AFFs) are uncommon complications of long-term use of bisphosphonates (BPs), a common medication used for osteoporosis and other conditions that reduce bone strength and density. BPs are effective in reducing the risk of fractures. Prolonged use of the medication can inhibit bone remodeling and increase the risk of insufficiency fractures, most especially in the subtrochanteric and diaphyseal regions of the femur. This case follows an 81-year-old Caucasian woman with a history of multiple myeloma (MM) in remission and long-term sodium clodronate use for skeletal protection, who was seen in the emergency department following a fall and severe right hip pain, preceded by two months of prodromal thigh pain. Initial imaging showed a comminuted fracture of the right femur with slight posterior displacement and an undisplaced atypical femoral fracture. Biochemical laboratory investigations revealed normal serum-adjusted calcium and phosphate with slightly low vitamin D levels. For a more precise diagnosis, the patient had a pelvic CT and pelvic MRI, which confirmed radiological features typical of AFFs, such as transverse fracture lines, cortical thickening, and minimal comminution, with no evidence of active myeloma recurrence. The patient's BP was controlled, and the AFF was managed with intramedullary nailing (IMN). This case emphasizes the importance of having a high index of suspicion in patients on long-term treatment with BPs to easily make the diagnosis of AFFs in elderly patients with prolonged BP exposure. Invariably, early recognition of AFFs will enhance timely intervention, which will prevent further complications of AFFs. Long-term BP therapy is beneficial in the management of osteoporosis; however, it increases the risk of developing AFFs. This case underscores the crucial need for patients to be aware of the signs of AFF, the importance of educating clinicians on how to diagnose AFFs, and the need for a multidisciplinary management approach in older patients on long-term treatment with BPs.

## Introduction

Atypical femoral fractures (AFFs) are usually termed insufficiency or stress-related fractures, which are associated with the long-term use of bisphosphonates (BPs) or antiresorptive bone medications [1]. Although the medications are useful for osteoporotic bones that are healthy, they hinder the healing of stress fractures, which may eventually cause a complete fracture [1]. Radiologically, AFFs have a transverse or short oblique configuration, with a localized periosteal or endosteal thickening along the lateral femoral cortex, and absence or minimal comminution [2,3]. Other features required for the diagnosis of AFFs include minimal or absence of trauma, prodromal pain, presence of comorbidities in the patients, and exposure to BPs, glucocorticoids, and other antiresorptive medications [2]. All these characteristics have been established by the American Society for Bone and Mineral Research (ASBMR) Task Force as major and minor criteria that can be used to identify AFFs [2].

BPs are widely used to treat osteoporosis in both men and women, most especially in postmenopausal women [4]. It works by inhibiting bone resorption by osteoclasts, which eventually increases bone mineral density and reduces the risk of fracture [5,6]. Although they have established clinical benefits, prolonged use of the medications, mostly after five years, has been shown to have serious side effects on the bones, which include AFFs [7,8]. The absolute incidence of AFFs ranges from 3.2 to 50 cases per 100,000 persons per year [1], but there is a significant increase in the relative risk when patients are more exposed to BPs, especially above five years [9].

The incidence of AFFs in patients under 65 years has been estimated to be 0.1-0.2 cases per 10 years; however, the incidence is higher in those over 65 years, 1.6 cases per 10,000 per year [8,9]. Moreover, among patients treated with BPs, the incidence of AFFs is estimated to be 3-10 cases per 10,000 patients per year [8,9]. Given this, recent clinical guidelines (such as those by the National Osteoporosis Guideline Group (NOGG)) now recommend a re-evaluation of BP use in patients who have been treated with BPs for a period of 3-5 years [10].

## Case presentation

An 81-year-old Caucasian woman with a past medical history of hypertension and multiple myeloma (MM) (in remission) presented to the hospital for a fall and with severe right hip pain. She admitted that she had been experiencing persistent pain in her right hip and upper thigh for the previous two months. She reported that on the day of her fall, while she was walking to complete some of her daily tasks, she felt an acute worsening of this pain, followed by the feeling that her right leg was giving way before she fell. She denied any preceding trauma that might have contributed to her fall. She was unable to weight bear following this incident. The patient was able to maintain independence with ambulation and activities of daily living before this and did not smoke/drink alcohol. She had no prior falls.

**Figure 1:**
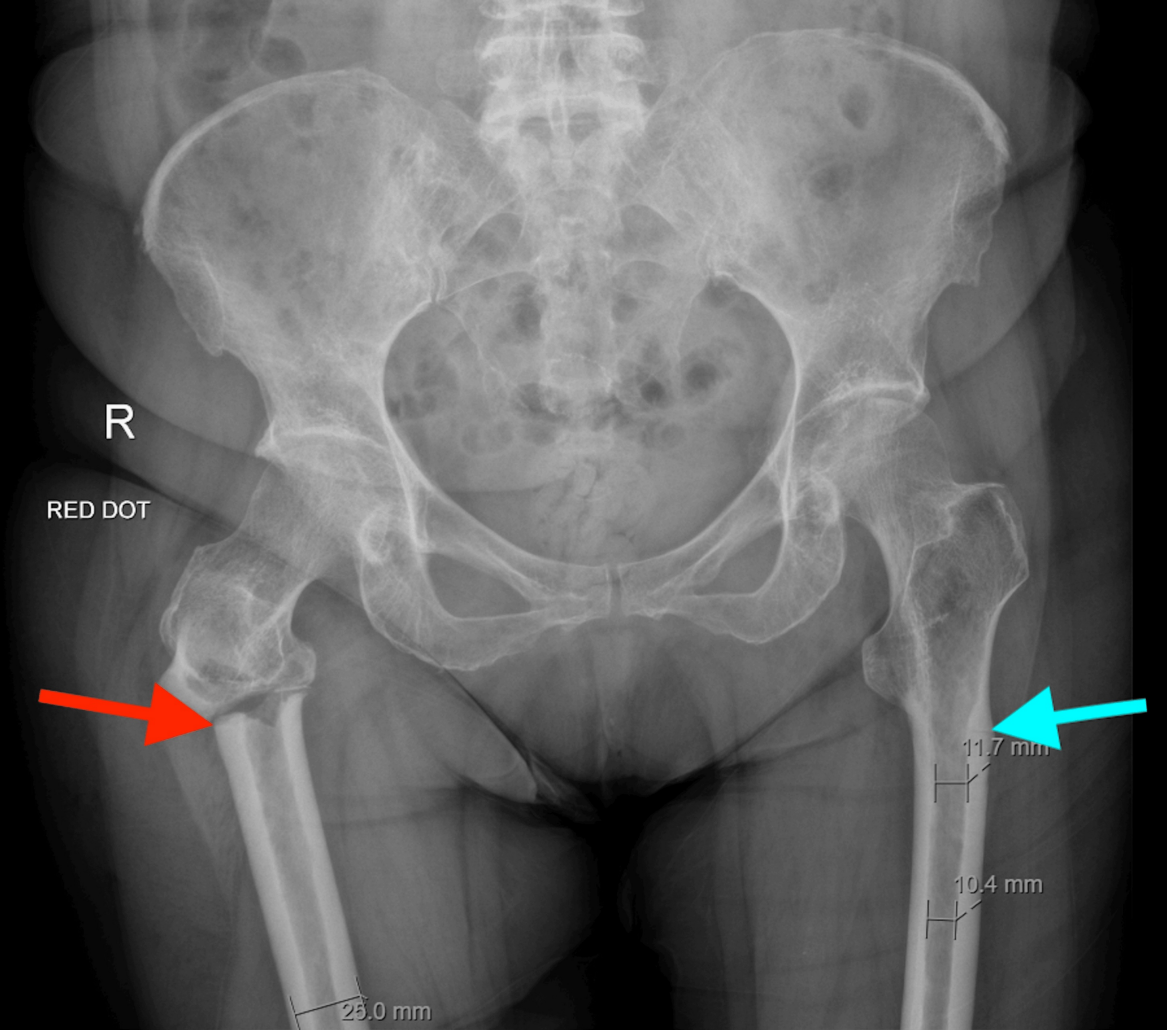
X-ray of the pelvis showing a suspected atypical fracture of the right femur There is a comminuted fracture of the proximal shaft of the right femur (red arrow) just inferior to the lesser trochanter with slight posterior displacement and angulation of the distal portion, likely an atypical fracture. Also noted was a transverse lucency with surrounding sclerosis seen within the outer cortex of the proximal femur on the left side at the lateral aspect (blue arrow).

On examination, she was found to have severe tenderness in the right hip and restricted range of motion of the right lower limb with erythema and swelling. Both lower limbs were found to be neurovascularly intact. She was otherwise hemodynamically stable, and the rest of the examination was unremarkable. A preliminary X-ray of her pelvis and bilateral hip revealed a comminuted fracture to the proximal shaft of the right femur just inferior to the lesser trochanter with slight posterior displacement and angulation of the distal portion; in addition, there was further transverse lucency with surrounding sclerosis seen within the outer cortex of the proximal femur on the left side at the lateral aspect; appearances were suggestive of an undisplaced atypical femoral fracture (Figure 1). She was then admitted to the orthogeriatric department for further management.

On further consideration of her past medical history, it was found that she had been diagnosed with multiple myeloma 11 years ago, for which she had received six cycles of cyclophosphamide, thalidomide, and dexamethasone (CTD). Additionally, she was also treated with an autologous stem cell transplant and radiotherapy. She had been receiving sodium clodronate for the last 10 years, as part of her management for complications of the multiple myeloma, such as skeletal metastasis and osteolytic lesions, as per her consulting hematologists. After her treatment, she remained in remission. She had a previous history of vertebral fractures, but none involving the hip joint.

**Figure 2:**
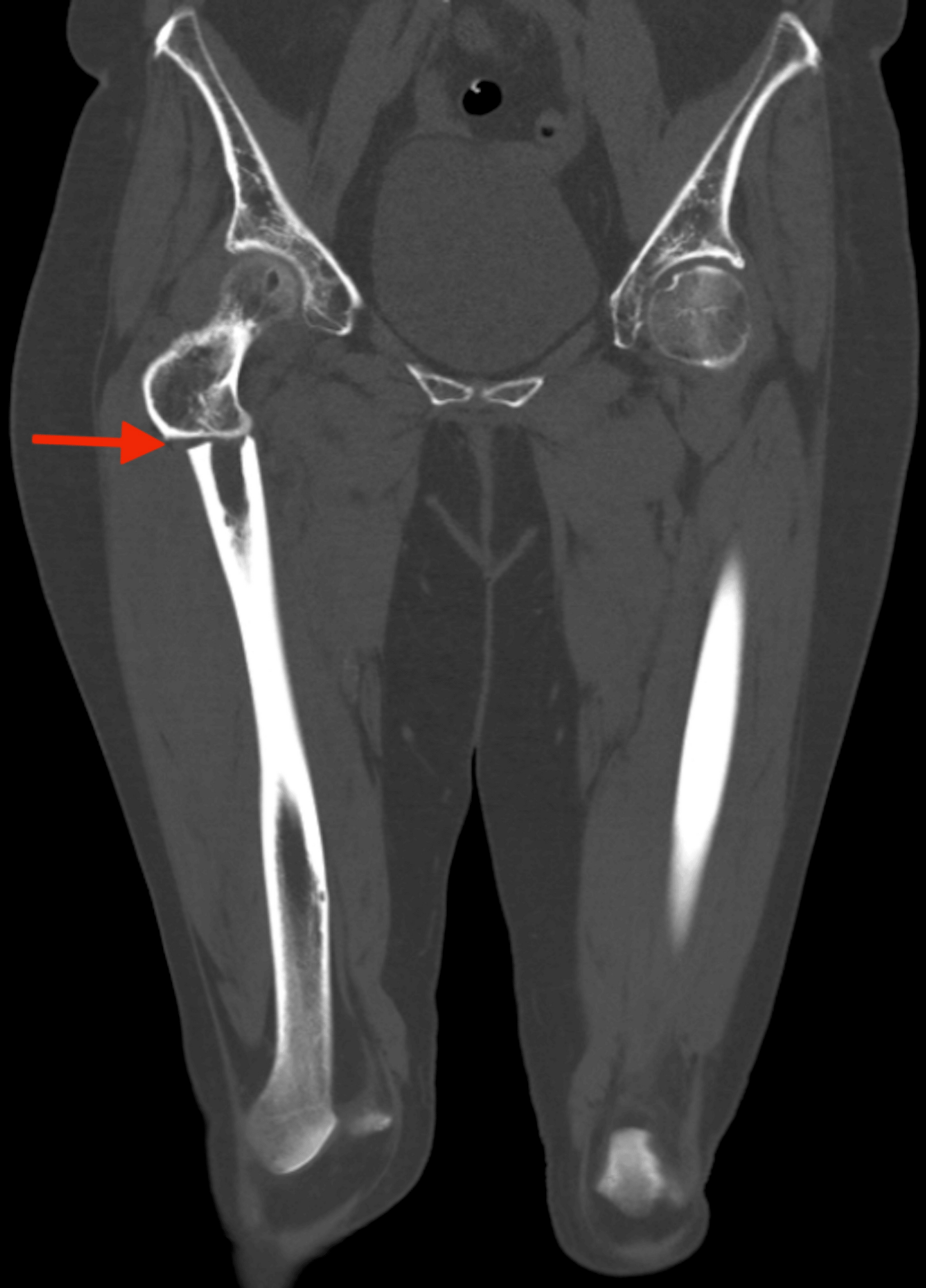
CT of the pelvis showing a displaced atypical fracture of the right femur The red arrow shows the atypical fracture of the right femur.

Laboratory investigations revealed that serum-adjusted calcium and phosphate were normal, with slightly low vitamin D levels (Table 1). A CT scan of the pelvis revealed a displaced right subtrochanteric neck of femur fracture. There was no significant overlying soft tissue subcutaneous swelling; however, there was increased attenuation and thickening of the quadriceps muscles, consistent with a recent injury. There was no acute fracture of the bony pelvis. There was subchondral sclerosis involving the inferior endplate of V5 vertebral body, which was suspicious for another inferior endplate fracture; there was also focal cortical lucency involving the anteromedial cortex of the right distal femur; within the bony pelvis, the bones of the sacral ala and the visualized lower lumbar spine appeared slightly radiolucent; given the past medical history, underlying myelomatous disease could not be excluded. They advised an MRI scan for further evaluation (Figure 2).

**Table 1:** Laboratory results showing normal serum-adjusted calcium and phosphate levels and mild vitamin D insufficiency

Parameter	Result	Reference range
Serum-adjusted calcium	2.54 mmol/L	2.20-2.60 mmol/L
Serum phosphate	1.23 mmol/L	0.8-1.5 mmol/L
Serum vitamin D	46.8 nmol/L	Normal: 50-75 nmol/L, insufficiency: 25-50 nmol/L, deficiency: <25 nmol/L

A preliminary myeloma screen was done (Bence Jones proteins and immunoglobulins), which was negative, and the patient was referred to hematology for further outpatient follow-up.

This case was discussed in a hip multidisciplinary team (MDT) meeting, and a decision was made to proceed with an intramedullary fixation of the right femoral fracture using a pediatric or adolescent nail due to the narrow canal, along with an MRI scan of her pelvis to assess for any impending fracture of the contralateral femur to consider prophylactic pinning if needed.

**Figure 3:**
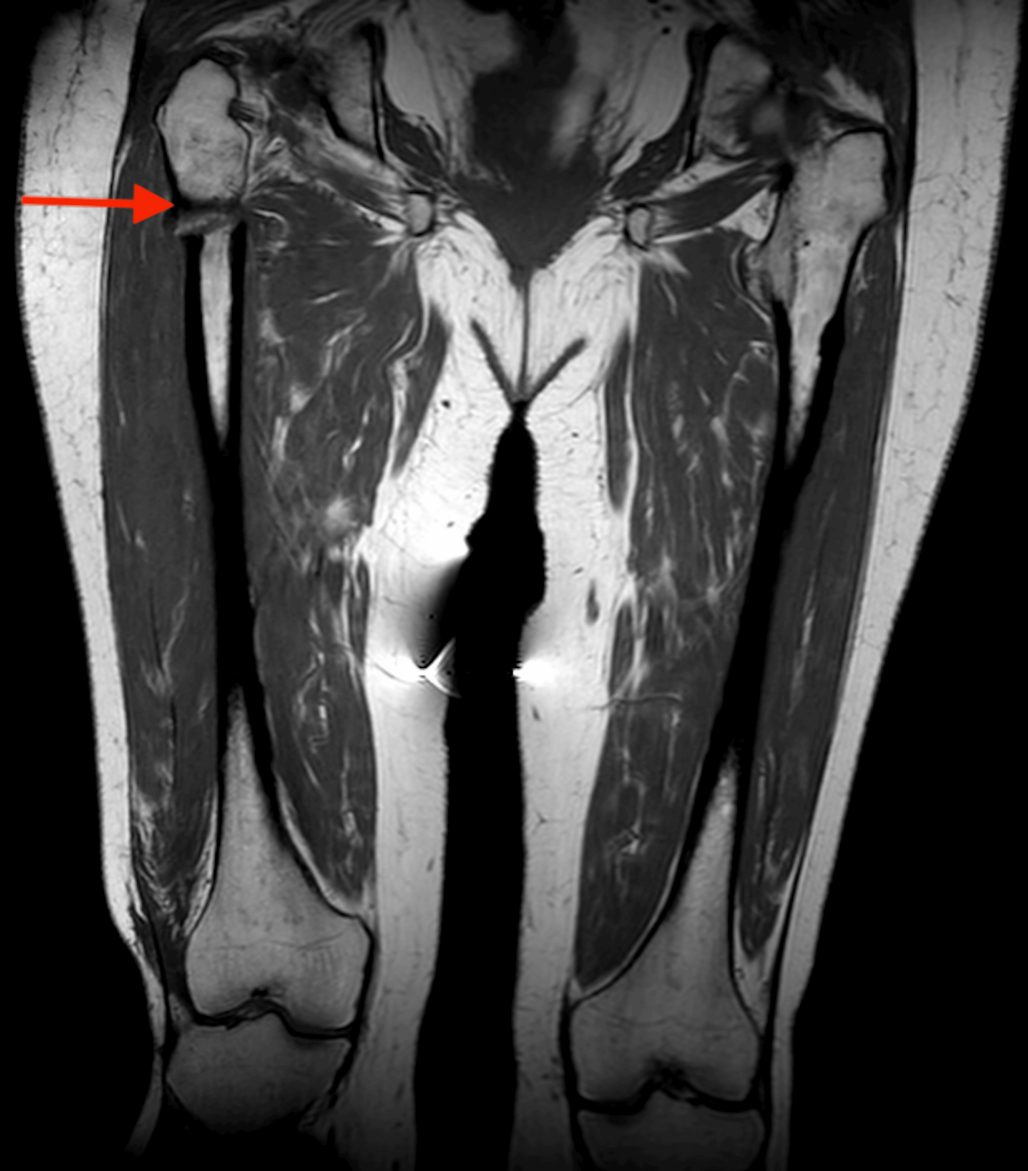
MRI of the pelvis showing a transverse fracture of the proximal right femur MRI of the pelvis exhibiting extensive transverse fracture line of the proximal right femur (red arrow) with moderately extensive adjacent muscle/soft tissue edema with sharp fracture margins and moderate cortical thickening along the proximal shaft of the left femur; there is no myeloma deposit; appearances are likely due to atypical fracture.

MRI of the pelvis was done, which exhibited extensive transverse fracture line of the proximal right femur with moderately extensive adjacent muscle/soft tissue edema with sharp fracture margins and moderate cortical thickening along the proximal shaft of the left femur; there was no myeloma deposit, and a conclusion was made that appearances are likely due to atypical fracture/bisphosphonate fracture (Figure 3). Given that the patient had no pain in her left lower limb, the orthopedic team decided that prophylactic pinning was not required at the time.

The patient was then discharged home with the required physiotherapy support and rehabilitation. She was also followed up in the fracture clinic in 10 weeks and was noted to be recovering satisfactorily. A dual-energy X-ray absorptiometry (DEXA) scan was done, which revealed a T-score of -2.1 at the left femur neck and -1.3 at the left femur in total, and a FRAX score (10-year absolute fracture risk) of 20.3% risk of major osteoporotic fracture and 6.5% risk of hip fracture. The report advised that the patient met the interventional threshold in line with NOGG guidelines; however, due to possible bisphosphonate-related femoral fractures and a history of multiple vertebral fractures, specialist opinion was recommended (Table 2).

**Table 2:** DEXA report highlighting only osteopenia in the left hip; however, as per NOGG guidelines, FRAX scores were at/above the intervention threshold Reference range: -1.0 or higher: healthy bone density, -1.0 to -2.5: osteopenia, -2.5 or lower: osteoporosis As per NOGG, the intervention threshold is a FRAX score of MOF of 20.3% or higher, and for hip fracture, a FRAX score of 4% or higher [10]. DEXA: dual-energy X-ray absorptiometry, NOGG: National Osteoporosis Guideline Group, FRAX score: 10-year absolute fracture risk, MOF: major osteoporotic fracture

Parameter	Site	Result
T-score	Left femur neck	-2.1
T-score	Left femur in total	-1.3
FRAX score	Major osteoporotic fracture	20.3%
FRAX score	Hip fracture	6.5%

She was later followed up by the rheumatology department, who advised her to cease the use of sodium clodronate entirely in light of her recent atypical fracture and indicated that she should remain off this medication unless specifically directed otherwise by them.

## Discussion

Multiple myeloma (MM) is a cancer that involves the abnormal growth of plasma cells. It is a serious disease ranging from a mild condition called monoclonal gammopathy of undetermined significance (MGUS) and potentially progressing to a more aggressive form such as plasma cell leukemia [11]. MM is either present outside the bone marrow as a solitary plasmacytoma or extramedullary plasmacytoma [11]. This condition is diagnosed when a specific type of abnormal protein (called M-protein) is found in the blood or urine, along with having more than 10% of bone marrow clonal plasma cells or a tumor made of these cells [11]. It also causes damage to organs or tissues that leads to high calcium levels in the blood, kidney problems, low red blood cell counts (anemia), and areas of bone loss [11]. This osteolytic lesion leads to bone pain, fractures, and sometimes compression of the spinal cord; for this purpose, bisphosphonates (BPs) are used [11]. They are structurally like inorganic pyrophosphates but are more stable and have a high affinity for calcium that target regions of high resorption on bone hydroxyapatite surfaces, in contrast to inorganic pyrophosphates [12].

In MM, osteoclast activity is abnormally increased due to cytokines (e.g., interleukin (IL)-6, IL-1, and IL-17) produced by interactions between myeloma cells and the bone marrow [13]. These cytokines stimulate bone resorption and inhibit bone formation. BPs and denosumab (DENOS), another bone-targeting agent, counteract this process by inhibiting receptor activator of nuclear factor kappa-B ligand (RANKL), a key mediator of osteoclast activity [13]. BPs inhibit the progression of osteoclastic activity and are therefore useful in the management of MM [13]. The importance of bisphosphonates in multiple myeloma has been proven in several studies [13]. In a German open-label study, patients treated with clodronate had fewer new bone lesions. Previous systematic reviews found that using bisphosphonates as part of myeloma treatment helps lower the chances of painful spine fractures and reduces pain for patients with multiple myeloma [14].

This case involves an 81-year-old woman with a significant past medical history of hypertension and multiple myeloma, previously treated with chemotherapy, autologous stem transplant, and radiotherapy. She had been in remission for myeloma for 11 years. She had been on sodium clodronate for 10 years for bone health, with a history of vertebral fractures, but no reported hip fractures. Imaging revealed a displaced subtrochanteric right femoral fracture likely related to long-term BP use. MRI confirmed no active myeloma but suggested atypical (BP) related fractures. DEXA scan indicated osteopenia (T-scores: -2.1 and 1.3), with FRAX scores showing elevated fracture risk. The rheumatologists advised permanently ceasing sodium clodronate, given the diagnosis of atypical fracture.

This case highlights multiple factors that support the diagnosis of atypical femoral fractures (AFFs). The history of long-term use of sodium clodronate along with the diagnostic imaging findings (transverse fracture lines, cortical thickening, and minimal trauma) strongly suggests BP-associated AFF.

In 2013, the American Society for Bone and Mineral Research (ASBMR) updated how atypical femoral fractures (AFFs) are identified, as shown in Table 3 [2,15]. These types of fractures usually occur in the area just below the hip (subtrochanteric region) or along the thigh bone (femoral shaft), often with little or no trauma [2]. It is important to tell them apart from more common fractures that result from serious injuries or those caused by bone tumors, which generally affect different anatomical locations of the femur, such as the neck of the femur or the intertrochanteric region [2]. AFFs tend to have a clean, simple break without many fragments (non-comminuted), and X-rays typically show a straight, horizontal break starting on the lateral side of the femur. Sometimes, the fracture extends across the bone to the inner (medial) side, giving it an oblique appearance [2]. A telltale sign of an impending AFF is a small, localized buildup of new bone, seen as "beaking" or "flaring," at the point where the fracture begins [2]. Recent studies have supported the use of ASBMR's criteria for identifying AFFs, although some features can be challenging to interpret [15]. Nonetheless, the presence of a lateral transverse fracture line with minimal fragmentation and evidence of new bone formation remains central to diagnosing AFF [2,15].

**Table 3:** ASBMR criteria for the diagnosis of atypical femur fractures ASBMR: American Society for Bone and Mineral Research Source: [2]

Major features	Minor features
The fracture is associated with minimal or no trauma, as in a fall from a standing height or less.	Generalized increase in cortical thickness of the femoral diaphysis.
The fracture line originates at the lateral cortex and is substantially transverse in its orientation, although it may become oblique as it progresses medially across the femur.	Unilateral or bilateral prodromal symptoms such as dull or aching pain in the groin or thigh.
Complete fractures extend through both cortices and may be associated with a medial spike; incomplete fractures involve only the lateral cortex.	Bilateral incomplete or complete femoral diaphysis fractures.
The fracture is non-comminuted or minimally comminuted.	Delayed fracture healing.
Localized periosteal or endosteal thickening of the lateral cortex is present at the fracture site ("beaking" or "flaring").	

In addition, the absence of recurrence of myeloma as confirmed on MRI helps rule out pathological fractures secondary to malignancy, supporting the diagnosis of BP causing the AFFs. Her DEXA scan results and elevated FRAX scores confirm compromised bone quality, although not severe enough to fully explain the fracture pattern without considering medication effects. Overall, this case illustrates the importance of recognizing AFFs in elderly patients with long-term bisphosphonate exposure, especially when presenting with prodromal thigh pain and minimal history of traumatic fractures.

The use of BP therapy is linked with serious side effects such as atypical femoral fractures (AFFs) and osteonecrosis of the jaw (ONJ). Therefore, to ensure the advantage of reducing the risk of fracture outweighs the risk of developing AFFs and ONJ, the National Osteoporosis Guideline Group has defined the optimal duration of BP therapy [10], as shown in Tables 4-6.

**Table 4:** High-risk groups requiring a longer course of oral bisphosphonates as per NOGG guidelines For oral BPs (such as alendronate, ibandronate, and risedronate), treatment should typically continue for at least five years, after which a person's fracture risk should be reviewed. However, a longer treatment period of 10 years or more is strongly recommended for certain individuals, including the above [10]. NOGG: National Osteoporosis Guideline Group, BP: bisphosphonate

High-risk groups requiring a longer course of oral bisphosphonates
People aged 70 or older when starting treatment
Those with a previous hip or spine fracture
People taking oral steroids (equivalent to 7.5 mg or more of prednisolone daily)
Those who suffer a fragility fracture during the first 5 years of bisphosphonate therapy, assuming no change is made to their treatment

**Table 5:** High-risk groups requiring a longer course of intravenous bisphosphonates as per NOGG guidelines For intravenous BPs (such as zoledronate), treatment should be given for at least three years, followed by a reassessment of fracture risk. A longer course of six years or more is strongly recommended for the same high-risk groups, such as above [10]. NOGG: National Osteoporosis Guideline Group, BP: bisphosphonate

High-risk groups requiring a longer course of intravenous bisphosphonates
People aged 70 or older at the start of treatment
Those with a history of hip or vertebral fractures
People on high-dose steroids (7.5 mg prednisolone or more per day)
Those who experience a fragility fracture during the first 3 years of treatment (if no change is made to the medication)

**Table 6:** NOGG guidelines on the further management of long-term BP therapy based on the recurrence of fractures This reassessment helps determine whether restarting treatment is necessary [10]. NOGG: National Osteoporosis Guideline Group, BP: bisphosphonate, FRAX score: 10-year absolute fracture risk

Recurring fractures	No recurring fractures
If someone stops taking bisphosphonates and then has a new fracture, their fracture risk should be re-evaluated using the FRAX tool, and treatment should be restarted (strong recommendation).	If no new fracture occurs after stopping treatment, the timing for reassessment depends on the type of bisphosphonate used: 18 months after stopping risedronate or ibandronate, 2 years after stopping alendronate, and 3 years after stopping zoledronate.

To promote bone health and reduce the risk of complications, a multidimensional approach is adopted to treat AFFs. In our patient's case, treating an AFF typically required surgical intervention using intramedullary nailing (IMN). Previous studies have outlined several reasons why intramedullary nailing (IMN) is generally favored over plate fixation for treating AFFs: mechanically, IMN offers better load-sharing and creates less bending stress due to its central position within the bone, unlike plate fixation, which sits on the outer surface [16]. This improved load distribution is especially beneficial for older patients, as it supports earlier mobility. Additionally, due to reduced bone remodeling and poor bone quality in these patients, there is a higher risk of stress fractures forming near the ends of implants [17]. Since it is challenging to cover the entire femur using plates, IMN provides an advantage by spanning the full length of the bone, and this allows for more stable fixation and a better balance of forces at the fracture site, potentially lowering the risk of future fatigue fractures compared to plating [18]. However, it is important to understand that non-union rates can be relatively high with these fractures [19]; therefore, it is vital to closely observe and follow up to check the progress of the fracture healing and to review if further intervention is required.

As demonstrated in this case, patients who are diagnosed with atypical femur fractures are at a high risk of developing contralateral hip fractures [20]. As a result, all patients with AFFs should undergo imaging of the contralateral hip to identify possible impending fractures that may require preventive treatment.

As high-risk individuals are more vulnerable to further fractures and complications, relying solely on a single reassessment at three or five years may be insufficient. Therefore, patients on extended bisphosphonate use should be routinely reviewed for pain in the thigh or groin region [21]. Those reporting pain should immediately undergo further diagnostic evaluation, such as bone mineral density (DEXA scan), FRAX, or other risk tools, with or without X-ray and MRI of the femur [21]. This should be done to monitor treatment efficacy, evaluate safety and side effects, and make timely decisions about treatment breaks or drug holidays, and operative fixation [21].

This case study provides valuable insights into a rare condition, contributing to the limited existing research on atypical femur fractures. It presents a detailed account of the diagnostic steps, treatment decisions, and patient outcomes, helping to improve understanding of how to manage such complex cases. However, as it focuses on a singular patient and lacks a comparison or control group, it is difficult to draw strong conclusions about the effectiveness of the treatment used. To confirm these observations and develop clearer treatment guidelines, larger studies involving more patients are necessary.

## Conclusions

AFFs, although initially thought to be a rare complication, are becoming increasingly prevalent in a specific cohort of patients. The early identification of prodromal signs in patients undergoing long-term BP therapy is crucial for significantly reducing the disease burden associated with AFFs. The underlying pathology indicates that while BP's anti-osteoclastic properties are beneficial in osteoporotic bones, they lead to an accumulation of micro stress fractures over time in healthy bones, eventually resulting in AFFs. This is why timely monitoring of BP therapy is paramount, as the NOGG guidelines state, ideally every 3-5 years. Furthermore, high-risk individuals must also be routinely reviewed for pain in the thigh or groin region and undergo further diagnostic investigations. Additionally, clinicians must anticipate possible impending contralateral AFFs in patients with a confirmed diagnosis of AFF; therefore, this must be explored using sensitive imaging, such as MRI, and prophylactic measures must be taken. Considering the delicate balance between the risks and benefits of long-term BP therapy, osteoporosis, and AFFs, specialist input should be sought when making definitive decisions. This case reinforces the critical need for consistent monitoring of BP therapy and offering comprehensive care in the management of atypical femoral fractures.
